# Exploring the Senotherapeutic Potential of Polyphenols in Aging and Disease: A Literature Review

**DOI:** 10.3390/ijms27083651

**Published:** 2026-04-19

**Authors:** Luna Braučič Mitrovic, Khrystyna O. Semen

**Affiliations:** University College Venlo, Campus Venlo, Faculty of Science and Engineering, Maastricht University, 5911 BV Venlo, The Netherlands; l.lbraucicmitrovic@alumni.maastrichtuniversity.nl

**Keywords:** fisetin, quercetin, senolytics, aging, nutrition, biomarkers

## Abstract

Aging is associated with an increased risk of developing many age-related diseases (ARDs), which are of major global health concern. In recent years, cellular senescence, characterized by cell cycle arrest and development of a senescence-associated secretory phenotype (SASP), has emerged as a key mechanism of aging and ARDs and has been increasingly explored as a promising therapeutic target. Among dietary bioactive ingredients, fisetin and quercetin have gained attention because of their potential to act as senolytics and senomorphics. This narrative literature review summarizes existing evidence exploring the potential of fisetin and quercetin to modulate senescence and SASP biomarkers in animal models of aging and progeria, as well as in interventional studies involving human subjects with geriatric syndromes and/or various ARDs. It also provides a brief overview of the molecular mechanisms of senescence and attempts to identify potential drivers and barriers for the clinical translation of those nutrients.

## 1. Introduction

Why and how we age remain among the most enduring scientific questions. Increasing attention is being directed toward whether the adverse health effects associated with aging can be mitigated. Aging occurs as a result of ongoing interactions between genetic factors and environmental influences, leading to the accumulation of cellular and biochemical damage [1]. Consequently, it is closely linked to an increased risk of developing various age-related diseases (ARDs), including neurodegenerative, cardiovascular and metabolic diseases, diabetes and cancer [2]. ARDs are the leading cause of death worldwide, with over 80% of individuals aged 65 and older experiencing at least one chronic condition, and 50% suffering from multimorbidity [3]. Therefore, the development of therapeutic approaches targeting ARDs is of utmost importance.

Over the last decade, cellular senescence has emerged as one of the most widely studied and accepted mechanisms underlying aging and ARDs. It is initiated by exposure to intrinsic and extrinsic factors causing DNA damage, replicative stress, increased production of inflammatory cytokines and accumulation of misfolded proteins [4,5,6]. Cells undergoing senescence (SCs) are characterized by irreversible cell cycle arrest and development of a senescence-associated secretory phenotype (SASP), which involves production of pro-inflammatory cytokines, growth factors, chemokines and proteases [4,7]. Supporting the critical role of cellular senescence in aging and ARDs, Baker et al. (2011) demonstrated that selectively eliminating SCs using a drug-inducible “suicide” gene delayed the onset of multiple ARDs and improved the health span in progeroid mice [8]. These findings suggested that selective elimination of SCs (senolysis) is essential for promoting healthy aging and preventing ARDs. In addition, modulating the functionality of SCs with inhibition of SASP (senomorphism) has also been recognized as a promising antiaging strategy [5].

The potential of food components to target senescence is of interest as it represents a safe, long-term strategy to support lifespan and health span [9]. Among dietary bioactives targeting senescence, fisetin and quercetin were identified as promising senotherapeutics. Fisetin (3,7,3′,4′-tetrahydroxyflavone) is a naturally occurring flavonol found in various fruits and vegetables, including strawberries (16.0 mg/100 g), apples (2.69 mg/100 g), onions (0.48 mg/100 g) and grapes (0.39 mg/100 g) [10,11,12]. The average dietary intake of fisetin in humans is estimated to be around 0.4 mg. It has been shown to have a variety of biological activities, including antioxidant, anti-inflammatory, anticancer, anti-hyperlipidemic, neuroprotective effects, etc. [11,12]. Accumulating evidence shows that fisetin can selectively reduce the viability and number of SCs in vitro and in vivo [12]. The primary mechanism responsible for this effect involves the induction of apoptosis through the inhibition of anti-apoptotic proteins (Bcl-2, Bcl-xl, BCL-W) and activation of pro-apoptotic factors such as Bcl-2-associated X (Bax) and Bcl-associated killer (Bak) [13]. This process leads to the release of cytochrome C, thereby inducing apoptosis in SCs. Fisetin was also shown to inhibit the signal transduction pathway PI3K/AKT, thus reducing SC survival [14]. Furthermore, its effect on senescence may also be attributed to its anti-inflammatory properties, namely, the downregulation of nuclear factor kappa B (NF-κB), which is a central regulator of SASP [15].

Quercetin (3,3′,4′,5,7-pentahydroxyflavone) is another bioactive flavonoid present in a variety of fruits and vegetables, including onions (4.5 mg/100 g), apples (4.0 mg/100 g), chili peppers (3.26 mg/100 g), kale (2.26 mg/100 g), cherries (1.74 mg/100 g) and asparagus (1.4 mg/100 g) [16]. High levels of quercetin have also been found in red wine (4–16 mg/L, depending on the type), tomato juice (13 mg/L), lemon juice (7.4 mg/L) and various black and green tea infusions (10–25 mg/L) [17]. Quercetin was the first natural compound identified with senolytic properties, which were attributed to its inhibitory effects on the PI3K pathway and serpins [18]. Similar to fisetin, quercetin was shown to have a direct inhibitory effect on Bcl-family proteins, triggering apoptosis [19]. Despite being shown to reduce signs of aging in an animal model when administered alone [20], the senotherapeutic potential of quercetin has been researched primarily for its combination with the tyrosine kinase inhibitor dasatinib (Q + D), which targets pro-survival AKT signaling, ephrin receptors and SCR kinases (EFNB1, EFNB3) [21]. The combination of Q + D was shown to eliminate a broader range of SCs compared to each compound administered alone, as dasatinib was found to be more effective in the elimination of human fat cell progenitors, while quercetin showed greater senolytic potential in senescent human endothelial cells [18]. Subsequent preclinical studies mainly focused on the ability of the Q + D cocktail to reduce SC burden, alleviate SASP expression, limit signs of aging and increase lifespan [22,23,24].

This narrative literature review summarizes existing evidence exploring the potential of fisetin and quercetin to modulate senescence and SASP biomarkers in animal models of aging and progeria, as well as in interventional studies involving human subjects with geriatric syndromes and/or ARDs. It also provides a brief overview of the molecular mechanisms of senescence and attempts to identify potential drivers and barriers for the clinical translation of those nutrients.

## 2. Mechanisms of Cellular Senescence

Cellular senescence has been identified as one of the interconnected hallmarks of aging, which, according to the updated framework introduced by López-Otín et al., 2023, also includes genomic instability, telomere attrition, epigenetic alterations, loss of proteostasis, disabled macroautophagy, deregulated nutrient-sensing, mitochondrial dysfunction, altered intercellular communication, stem cell exhaustion, chronic inflammation and dysbiosis [1]. SCs are characterized by permanent cell cycle arrest, resistance to apoptosis and activation of certain intracellular signaling pathways [25]. Cellular senescence is different from other forms of cell quiescence (G0), as it occurs in the G1 and possibly G2 phases of the cell cycle and is irreversible [26]. Furthermore, transition to this state develops over longer periods of time (weeks in cell culture), which differs from other situations leading to a switch to G0 [27].

The main pathways involved in transition to the senescent state are the p16^INK4A^–/retinoblastoma protein (RB) pathway and the p53/p21^Cip1^ pathway [28]. More specifically, cyclin-dependent kinase (CDK) inhibitor p16^INK4a^ blocks CDK4 and CDK6, while p21^Cip1^ inhibits CDK2, resulting in hypophosphorylation of RB and subsequent replicative arrest [4]. SCs also display resistance to apoptosis by upregulating anti-apoptotic members of the B cell lymphoma 2 (BCL-2) family, including BCL-2, BCL-XL and BCL-W, leading to sequestration of pro-apoptotic factors via inhibition of permeabilization of the outer mitochondrial membrane [4]. Modulation of NF-kB signaling was shown to be involved in the initiation of growth arrest and maintenance of the senescence phenotype [29]. Genotoxic stress and DNA damage, which are recognized etiological factors of aging, trigger signaling cascades involving PARP-1 and ataxia telangiectasia mutated kinase (ATM), which interact with the IKK complex and trigger NF-kB activation [29]. In SCs, upregulation of NF-kB is instrumental in the maintenance of senescence-specific secretome and is supported by activation of p38MAPK kinases, upregulation of the PI3/AKT pathway and loss of inhibition by SIRT1 [5]. Furthermore, persistent activation of the nutrient-sensing signaling pathway PI3K/AKT/mTOR (mammalian target of rapamycin) supports the development of distinct morphological changes, such as cell enlargement and flattening, increased activity of senescence-associated β-galactosidase (SA-β-gal), a decrease in lamin B1 [5], as well as maintenance of a metabolically active state [30].

Within a pool of cells undergoing senescence, 30–70% start to produce a variety of biomolecules, the so-called SASP factors. These include inflammatory cytokines, such as tumor necrosis factor-alpha (TNF-α), interleukin (IL)-6 and IL-1β; chemokines, such as IL-8 and chemokine (C-X-C motif) ligand (CXCL)2; and matrix metalloproteinases (MMPs); proteases, such as plasminogen activator inhibitor (PAI)-1 and growth factors, e.g., growth differentiation factor (GDF)-15 [9,19,28]. Production of SASP factors further promotes cellular senescence via autocrine and paracrine mechanisms [31]. SASP factors were shown to be upregulated in animal models of aging and ARDs, while administration of SASP inhibitors, also defined as senomorphics, showed promise in the management of ARDs [32].

Another major contributor to the senescence phenotype is senescence-associated mitochondrial dysfunction (SAMD), which involves excessive production of reactive oxygen species and decreased mitochondrial membrane potential [33,34]. SAMD results from upregulation of p38MAPK- and TGFβ-dependent pathways [33,34] and/or activation of NF-kB [35], supporting the development of SASP. Furthermore, increased production of reactive oxygen species by dysfunctional mitochondria contributes to DNA damage and epigenetic modifications, such as changes in chromatin, DNA methylation, and histone alterations, further supporting the mechanisms of cellular senescence [35,36].

## 3. Fisetin and Quercetin in Animal Models of Aging

### 3.1. Study Design

To analyze the potential of fisetin and quercetin to target aging in vivo, we searched for articles that tested the effects of those nutrients in various models of aging and assessed at least one senescence biomarker as an outcome. In total, 18 studies were identified, of which six assessed the effects of fisetin and twelve assessed the effects of quercetin in combination with dasatinib (Q + D) (Table 1). The majority of studies used mouse models, among which naturally aging C57BL/6 mice were the most frequent. Several studies employed progeroid SAMP10 and Ercc1^−/Δ^ mouse models, which offer a time-efficient alternative to natural aging or chemically induced premature aging models (Table 1). Only one study was conducted in large animals (old female sheep), making it of special interest for translation to humans [37], as mouse models of aging are recognized with certain limitations for the development of translationally relevant measures and biomarkers characterizing human aging [38]. Overall, the heterogeneity in the animal models used presents a certain challenge for the interpretation of the results due to potential differences in the rate of aging and tissue distribution of SCs.

Fisetin was administered via oral gavage or dietary supplementation in all studies except the one conducted in large animals, which used intravenous injections [37]. Doses of fisetin ranged from 50 to 100 mg/kg and were given daily or in intermittent cycles (e.g., 2–3 consecutive days every two weeks) over periods from one day to four months. Interestingly, studies performed in various cell types confirmed its senolytic potential based on the ability to reduce viability and SC number and modulate the expression of CDK inhibitors [12], which favors an intermittent dosing approach. At the same time, continuous daily administration of fisetin was also shown to be efficient and safe [39].

Quercetin was mostly administered in combination with dasatinib, except for in the study by Xing et al. (2023), which tested the effects of quercetin alone using a liposome delivery system to improve the bioavailability of this bioactive [40]. The standard combination included 50 mg/kg of quercetin and 5 mg/kg of dasatinib administered via oral gavage [23,24,41,42,43,44,45] or intraperitoneal injections [46,47] intermittently for 3 consecutive days every 2 weeks over 1–3 months (Table 1). The effects of biweekly four-month [24,42] and five-month [46] uses were also tested. The intermittent (“hit-and-run”) treatment was mostly used, as it is a well-established approach for testing the senolytic effects.

The reviewed animal studies employed a broad range of senescence markers and SASP factors. To evaluate the senolytic effect, cell cycle arrest biomarkers p16^INK4a^ and p21^Cip1^ were most commonly measured, followed by assessment of SA-β-gal activity [18,37,39,41,42,48] and p53 expression [37,49,50] (Table 1). Selection of SASP factors varied significantly between studies, with IL-1β, IL-6 and MCP-1 being the most common [23,24,39,41,42,43,46,47], followed by TNF-α [23,39,43,47,51], CXCL2 and CXCL10 [23,43,46], as well as IL-10 [37,39,43,52]. Other measured SASP factors included IL-1α, IL-8, CXCL10, and PAI-1.

P21^Cip1^ is a downstream effector of p53 and a member of the Cip/Kip family of CDK inhibitors, which has emerged as a promising candidate to be a universal biomarker of SCs [53]. In addition, it is recognized to be crucial for various cellular processes, including differentiation, DNA repair, transcription and migration [54]. Research showed that the number of p21^Cip1^-positive cells increases with age, but this response is organ-specific, as it was noted in the skin, pancreas, and kidney, but not in the lungs [55]. Compared with another biomarker of senescence, p16^INK4a^, p21^Cip1^-positive cells constitute a distinct cell population characterized by differences in cell type, tissue distribution, accumulation kinetics, and physiological effects [56]. A recent review analyzed the contribution of p21^Cip1^ upregulation to different senescence phenotypes and diseases associated with aging and pointed out the close links between its expression in adipose tissue and progression of metabolic and cardiovascular disease [53].

Another CDK inhibitor, p16^INK4a^, has emerged as a systemic aging marker, as it has been shown to be upregulated with advances in age across various tissues, while the clearance of p16-expressing cells was shown to delay age-associated disorders and extend median lifespan [8,57]. Activation of P16^INK4a^ is a cellular stress response that occurs in multiple organs and tissues and stops a cell from advancing from the G1 phase to the S phase. P16^INK4a^ expression is recognized to reflect biological age and is perceived as a predictor of toxicity in patients treated with chemotherapy [57]. Increased expression of p16^INK4a^ was implicated in the development of pulmonary fibrosis due to its association with persistent profibrotic signaling, fibroblast activation, and extracellular matrix deposition [58]. Together with p21^Cip1^, upregulation of p16^INK4a^ has been implicated in cardiac aging, and reducing its expression has been shown to protect against heart remodeling and dysfunction [53].

### 3.2. Effects on Senescence and Aging

The therapeutic efficacy of studied nutraceuticals was assessed by measurement of senescence biomarkers and, in some cases, evaluation of the clinical signs of aging. The capacity of fisetin to target SCs was shown in in vivo models by its capacity to reduce the percentage of SA-β-gal+ cells, as well as *p16^INK4a^* and *p21^Cip1^* cells, in various organs and tissues [37,39]. Acute, chronic/intermittent administration of fisetin was found to reduce *p16^INK4a^* expression in the spleen, liver, kidney, fat and aorta of aged C57BL/6 mice and also in the old sheep model [37,39,51]. The study by Harrison et al. (2024), which used UM-HET3 mice, did not show any effects [52]. The expression of CDK inhibitor p21^Cip1^ was reduced after acute and long-term use of fisetin in the spleen, liver, kidney, fat and aorta of aged mice [37,39,49,50]. Moreover, Yousefzadeh et al. (2018) demonstrated that acute and long-term supplementation with fisetin reduced various SASP factors (*IL-1β*, *IL-6*, *IL-10*, *TNF-α*, *CXCL2*, *MCP-1*, and *PAI-1*) in the CD3+ T cells, spleen, fat, liver, and kidneys of naturally aged and progeroid mice [39]. In contrast, Mahoney et al. (2024) demonstrated no effect of intermittent fisetin administration on *TNF-α* and *CXCL2* expression, while reduced expression of *CCL2* and *MMP3* was noted [51]. Overall, supplementation with fisetin was shown to reduce SC burden and to ameliorate SASP factor production in animal models of aging.

Similarly, quercetin alone or in combination with dasatinib was found to modulate senescence and SASP biomarkers in animal models of aging (Table 1). A consistent finding is that the combination of Q + D significantly reduced p16^INK4a^ expression in various tissues and organs, including adipose tissue, liver, hippocampus, intervertebral discs, cartilage, muscle and cardiac muscle [18,23,24,41,42,44,45,46,47]. Additionally, use of Q + D caused a significant reduction in p21^Cip1^ expression in the hippocampus, endothelial tissue, adipose tissue and intervertebral discs [23,41,45,46,48]. The administration of quercetin alone demonstrated similar findings, with reduced p16^INK4a^ and p21^Cip1^ expression in femur; however, the evidence remains sparse, with only one study to date examining the independent effect of quercetin in animals [40]. SA-β-gal activity also decreased significantly in the fat tissue, muscle and hippocampus of male mice following Q + D treatment [18,41,42,46].

**Table 1:** Studies exploring the potential of fisetin and quercetin to modulate senescence biomarkers in aged and progeroid animal models.

Animal Model	Age and Sex at Time of Treatment	Route of Administration	Dose	Frequency and Duration	Senescence Biomarker	Results	Ref.
**Fisetin**							
**Old UM-HET3 mice**	20-month-old male and female	Oral, supplementation in chow diet	600 ppm	Daily	*p16^INK4a^* expression	FIS did not reduce *p16^INK4a^* in the liver, kidney, or brain.	[52]
				3 consecutive days every 2 weeks for 2–4 months			
**Old Columbia Cross Sheep**	6–7-year-old female	Intravenous injections	100 mg/kg FIS or vehicle	2 consecutive days per week for 2 months	p16^INK4a^+ cells, *p21^Cip1^*, *p53*, *IL-10*, *IL-8* expression and SA-β-gal+ cells	FIS significantly reduced the number of p16^INK4a^-positive neurons, astrocytes, and microglial cells in the cerebral cortex; the number of SA-β-gal-positive cells in the brain cortex and grey matter of the cerebellum; *p21^Cip1^* expression in lungs and liver; and *IL-10* expression in liver compared to V. FIS nonsignificantly decreased *p21^Cip1^* expression in brain cortex and *p53* expression in the lung and liver. FIS had no effect on p16^INK4a^-positive cells in the hippocampus; *IL-8* and *IL-10* expression in the brain cortex, spleen tissue, lung and bone marrow; *p21^Cip1^* and *p53* expression in the heart and spleen tissues and brain cortex; or *p21^Cip1^* expression in bone marrow compared to V. FIS increased *p53* expression in bone marrow; *IL-8* and *IL-10* expression in heart tissue; and *IL-8* expression in the liver compared to V.	[37]
**Old C57BL/6 mice**	16.5-month-old male	Oral gavage	50 mg/kg FIS or vehicle	Daily for 1 week	p21^Cip1^ and p53 expression	FIS significantly reduced p21^Cip1^ and p53 in the aorta compared to V.	[49]
**C57BL/6 mice W Adriamycin-** **induced premature aging**	2-month-old male	Oral gavage	50 mg/kg FIS or vehicle	Daily for 1 month	p21^Cip1^ and p53 expression	FIS significantly reduced p21^Cip1^ and p53 in the aorta compared to V.	[50]
**Old C57BL/6 ** **mice**	27-month-old male	Oral gavage	100 mg/kg FIS or vehicle	Once daily for a week, followed by 2 weeks with no dosing for 2 cycles	p16^INK4a^, *TNF-α*, *CCL2*, *CXCL2*, *MMP-3* and *VEGF* expression	FIS significantly reduced p16^INK4a^, *CCL2*, and *MMP−3* expression in the aorta compared to V. FIS did not affect *TNF-α*, *CXCL2* and *VEGF* expression in the aorta compared to V.	[51]
**p16^+/Luc^; Ercc1^−/∆^ progeroid mice**	1.5-month-old male	Oral, supplementation in chow diet	≈60 mg/kg FIS or normal chow diet	Daily for 2 weeks, between 6 and 8 and 12 and 14 weeks of age	*p16^INK4a^* expression	FIS significantly reduced the level of *p16^INK4a^* expression during treatment and for four weeks post-treatment (weeks 8 to 12) compared to the control group.	[39]
**Ercc1^−/∆^** **progeroid mice**	2.5-month-old male and female	Oral, supplementation in chow diet	≈60 mg/kg FIS or normal chow diet	Daily for 10 weeks	*p16^INK4a^*, *p21^Cip1^*, *IL-1β*, *-6*, *-10*, *TNF-α*, *CXCL2*, *MCP-1* and *PAI-1* expression	FIS significantly reduced *p16^INK4a^*, *p21^Cip1^*, *IL-1β*, *-6*, *-10*, *TNF-α*, *CXCL2*, *MCP-1*, and *PAI-1* in CD3+ T cells, spleen, fat, liver, and kidneys compared to the control group.	
**Old C57BL/6 mice**	22–24-month-old male	Oral gavage	100 mg/kg FIS or vehicle	5 consecutive days	p16^INK4a+^ cells and SA-β-gal+ inguinal fat cells	FIS significantly reduced SA-β-gal+ cells in inguinal fat and the number of p16^INK4a^-expressing stem/progenitor, T lymphocytes, NK and endothelial cells compared to V. FIS had no effect on the number of p16^INK4a^ expressing macrophages or dendritic cells compared to V.	
**Old f1 C57BL/6: FVB mice**	21-month-old male and female	Oral supplementation in chow diet	60 mg/kg FIS or normal chow diet	Daily for a lifetime	*p21^Cip1^*, *IL-1β*, *-6*, *-10*, *TNF-α*, *CXCL2*, *MCP-1*, and *PAI-1* expression	FIS significantly reduced *p21^Cip1^*, *IL-1β*, *-6*, *-10*, *TNF-α*, *CXCL2*, *MCP-1*, and *PAI-1* in CD3+ T cells, spleen, fat, liver, and kidneys compared to the control group.	
**Quercetin**							
**Old C57BL/6 mice**	22-month-old female and male	Oral gavage	50 mg/kg Q + 5 mg/kg D or vehicle	3 consecutive days every 2 weeks for 2 months	*p16^INK4a^*, *p21^Cip1^*, *IL-1β*, and *-6*, expression and SA-β-gal+ cells	Male mice: Q + D significantly reduced *p16^INK4a^* and *IL-6* expression and SA-β-gal+ cells; insignificantly decreased *IL-1β*; and had no effect on *p21^Cip1^* expression in the hippocampus compared to V. Female mice: Q + D significantly reduced *p21^Cip1^* expression, while there was no effect on *p16^INK4a^*, *IL-1β*, *-6* and SA-β-gal+ cells in the hippocampus compared to V.	[41]
**Old C57BL/6 mice**	21-month-old male	Oral gavage	50 mg/kg Q + 5 mg/kg D or vehicle	3 consecutive days every 2 weeks for 3 months	*p21^Cip1^* expression in carotid artery endothelial cells	Q + D significantly reduced endothelial *p21^Cip1^* expression compared to V.	[48]
**Old C57B1/6 mice**	20-month-old male	Oral gavage	50 mg/kg Q + 5 mg/kg D or vehicle Bacl2 injection 7 or 28 days prior to euthanasia	Every 2 weeks for 4 months	*p16^INK4a^* expression, serum IL-1α, -1β, -6, and MCP-1 and SA-β-gal+ cells	Q + D significantly reduced *p16^INK4a^* expression in the muscle of 7D Bacl2 mice and SA-β-gal+ cells in the muscle of 28D Bacl2 mice compared to V. Q + D significantly reduced serum MMP3 compared to V. Q + D had no effect on the number of SA-β-gal+ cells in the muscle of 7D Bacl2 mice compared to V. Q + D had no effect on serum IL-1α, -1β, -6, and MCP-1 compared to V.	[42]
**Old C57BL/6 mice**	21-month-old male	Oral gavage	50 mg/kg Q + 5 mg/kg D or vehicle	3 consecutive days every 2 weeks for 3 months	p16^INK4a^ and *p21^Cip1^*, *IL-1α*, *-1β*, *-6*, *TNF-α*, *CXCL2*, *CXCL10* and *MCP-1* expression	Q + D significantly reduced *p16^INK4a^* and *MCP-1* expression in pgWAT and liver and *p21^Cip1^*, *IL-1α*, *-1β*, *-6*, *TNF-α*, *CXCL2*, *CXCL10* expression in pgWAT compared to V. Q + D had no effect on *p21^Cip1^*, *IL-1β* or *TNF-α* expression in skeletal muscle and liver compared to V.	[23]
**Old Wistar rats**	22-month-old (sex not specified)	Oral gavage	50 mg/kg Q + 5 mg/kg D or vehicle	5 days per week every 2 months	IL-1α, -1β, -2, -4, -5, -6, -10, -12, -13, -17A, -18, TNF-α, CXCL10, MCP-1, MIP1a, MIP2, G-CSF, GM-CSF, VEGF in serum	Q + D significantly reduced serum IL-1α, -1β, -2, -4, -5, -6, -10, -12, IL-13, -17A, -18, TNF-α, CXCL10, MCP1, MIP1a, MIP2, G-CSF, GM-CSF and VEGF compared to V.	[43]
**Old C57BL/6 mice**	14-month-old female and male	IP injections	50 mg/kg Q + 5 mg/kg D or vehicle	Once weekly for 5 months	p16^INK4a^, p19^ARF^, p21^Cip1^, IL-1β, -6, -15, -16, -17A/F, -17E, -21, -22, -27 -31, -33, CXCL10, MCP-1, MIP-1α, MCP-2 and MMP13	Q + D significantly reduced p16^INK4a^, p19^ARF^, p21^Cip1^, IL-6 and MMP13 and increased IL-1β expression in intervertebral discs compared to V. Q + D significantly reduced IL-1β, -16, -17E, -21, -22, and -31, with no effect on IL-15, -17A/F, -27p28/IL-30, -33, CXCL10, MCP-1, MIP-1α and MCP-2 plasma levels compared to V.	[46]
**Old C57BL/6 mice**	18-month-old female and male	IP injections	50 mg/kg Q + 5 mg/kg D or vehicle	Once weekly for 5 months		Q + D significantly reduced IL-1β plasma, while there was no effect on p16^INK4a^, p19ARF, and RB expression in intervertebral discs and plasma IL-15, -16, -17A/F, -21, -22, -27p28/IL-30, -31, -33, CXCL10, MIP-2, MIP-1α, and MCP-1 levels compared to V.	
**SAMP10 mice**	7.5-month-old male	Oral gavage	50 mg/kg Q + 5 mg/kg D or vehicle	3 consecutive days every 2 weeks for 2 months	p16^INK4a^ expression	Q + D significantly reduced p16^INK4a^ expression in the hippocampus, with no effect in the soleus muscle compared to V.	[44]
**Old C57BL/6 mice**	20–24-month-old female	IP injection	50 mg/kg Q + 5 mg/kg D or vehicle	3 consecutive days every week for 1 month	p16^INK4a^, IL-1α, -1β, -6, -8, TNF-α, CCL11, MMP3, MCP-1, PAI-1 and GM-CSF, expression	Q + D significantly reduced positive p16^INK4a^ CM and nonmyocyte cardiac cells; IL-1α, -1β, -6, CCL11 and PAI-1 expression in CMs; and IL-1α, -1β, -6, -8, TNF-α, CCL11, MMP3, MCP1, PAI-1 and GM-CSF expression in nonmyocyte cardiac cells compared to V.	[47]
**Old C57BL/6 mice**	24-month-old male	Liposomes via intravenous injection or IP injection	150 uL Q	Three times a week	p16^INK4a^ and p21^Cip1^ expression	Both methods significantly reduced p16^INK4a^ and p21^Cip1^ expression in femurs, while liposome treatment resulted in a greater reduction in both mouse models compared to IP injection.	[40]
**C57BL/6 doxorubicin-induced-senescence mice**	3-month-old male						
**Old C57BL/6 mice**	20-month-old male and female	Oral gavage	50 mg/kg Q + 5 mg/kg D or vehicle	Once every 2 weeks for 4 months	*p16^INK4a^*, *IL-6*, *CXCL1* and *MCP-1* and *CXCL1* expression	Q + D significantly reduced *p16^INK4a^*, *IL-6* and *CXCL1* expression in visceral adipose tissue compared to V, while no effect was seen on *MCP-1* expression.	[24]
**Old C57BL/6 mice**	23-month-old male and female	Oral gavage	50 mg/kg Q + 5 mg/kg D or vehicle	3 consecutive days every two weeks for 1.5 months	p21^Cip1^ and MMP13 expression	Q + D significantly reduced p21^Cip1^ MMP13 expression in cartilage compared to V.	[45]
**Old C57B1/6 mice**	>24-month-old male	Oral gavage	50 mg/kg Q + 5 mg/kg D or vehicle	Once	*p16^INK4a^* expression and SA-βgal+ cells	Q + D significantly reduced number of *p16^INK4a^*-expressing cells in fat and liver, and SA-βgal+ cells in fat compared to V.	[18]

The administration of quercetin and Q + D combination attenuated the production of SASP factors (Table 1). For example, IL-6 was found to significantly decrease post-treatment in various tissues and organs, including cardiac myocytes, perigonadal white adipose tissue (pgWAT), hippocampus, intervertebral discs and plasma [23,41,43,46,47]. The same studies showed a reduction in IL-1β in serum, plasma, myocytes, nonmyocyte cardiac cells and the male hippocampus. In contrast, no effect of Q + D treatment on IL-1β levels was found in the liver and muscle [23], female hippocampus [41] or serum [42]. IL-1α expression was found to be significantly decreased in pgWAT [23], serum [43], myocytes and nonmyocyte cardiac cells [47]. Conversely, Dungan et al. (2022) found no effect of Q + D on serum IL-1α [42]. Effects on MCP-1 expression were inconsistent. Some studies illustrated a significant decrease post-treatment in liver, pgWAT and plasma levels [23,46], while others found no significant effect [24,42,46]. Lastly, three studies illustrated a significant reduction in TNF-α expression in pgWAT [23], serum [43] and non-cardiomyocytes [47], while no effect was found in skeletal muscle and the liver [23]. Overall, Q + D consistently reduced the expression of senescence markers (p16^INK4a^ and p21^Cip1^), SA-β-gal activity and modified the production of IL-1α, IL-1β, IL-6, MCP-1 and TNF-α in aged and progeroid mice. The effects, however, appear to be influenced by sex, age and tissue type.

Beyond senolytic effects, administration of Q + D has also been shown to improve clinical outcomes in mice. Zhu et al. (2015) demonstrated that Q + D treatment in Ercc1^−/Δ^ mice significantly alleviated age-related symptoms (including tremors, loss of grip, ataxia and impaired gait) and extended lifespan [18]. Supporting and extending these findings, subsequent studies reported that Q + D alleviated age-related frailty, muscle weakness and osteoporosis [18,22]. Additionally, Ota and Kodama (2022) illustrated that Q + D treatment supported cognitive function during aging [44]. These improvements in clinical outcomes are likely driven by reduced SC burden and inhibition of SASP factors. Fisetin was shown to reduce age-related pathology and extend median and maximum lifespan in old C57BL/6 mice [39]. However, no effect on lifespan was observed in old UM-HET3 mice [52].

## 4. Fisetin and Quercetin in Human Research

### 4.1. Study Design and Selection of Senescence Biomarkers

We identified four clinical studies that investigated the effects of nutrients of interest and assessed changes in biomarkers of senescence (Table 2). All studies were phase 1 or pilot studies that used intermittent administration of Q + D and followed a single-arm, open-label design. They enrolled individuals with various pathologies, including early-stage Alzheimer’s disease or mild cognitive impairment (AD/MCI) [59,60], chronic diabetic kidney disease (CDKD) [61] and idiopathic pulmonary fibrosis (IPF) [62]. Dasatinib was administered at 100 mg/day and quercetin at 1000–1250 mg/day (Table 2), either for 2–3 consecutive days [61] or following an intermittent dosing strategy for 3 or 12 weeks [59,60,62]. None of the studies was powered to assess the efficacy of the tested interventions. A broad panel of senescence biomarkers and SASP factors was measured in blood, plasma, cerebrospinal fluid, adipose tissues and the epidermis (Table 2). The senescence markers p16^INK4a^ and p21^Cip1^, which are most commonly used in animal models, were assessed in patients with CDKD [61] and early-stage AD [60]. Changes in SA-β-gal activity in adipose tissue were measured in diabetic individuals [61]. IL-6 was the most frequently measured SASP factor, followed by IL-1α, IL-8, TNF-α, MMP-2 and MMP-9 (Table 2).

### 4.2. Effects on Senescence and Relevant Clinical Outcomes

Current findings on the senolytic effects of Q + D treatments show significant heterogeneity, which can be explained by the differences in study designs and target populations. Interestingly, all identified studies (Table 2) reported Q + D to be safe and well tolerated. The open-label pilot study in IPF patients demonstrated reduced IL-7 and TIMP2, while no significant effects were observed on other tested SASP factors after the 3-week Q + D treatment [62]. The clinical effects in this population involved a significant improvement in physical function (6-min walk, gait speed, chair stands, short physical performance battery), though no changes were observed in lung function, fatigue or quality of life. Interestingly, some correlations between the SASP factors and indices of pulmonary function were found [62]. A subsequent randomized controlled study demonstrated good tolerance and feasibility of intermittent Q + D treatment in IPF patients [63]. The study, however, did not assess changes in senescence biomarkers except for the novel biomarker glycoprotein nonmetastatic melanoma protein B (GPNMB or osteoactivin), which did not change significantly compared with placebo [63].

In CDKD patients, a significant reduction in p16^INK4a^- and p21^Cip1^-positive cell abundance was observed in adipose tissue 11 days after 3-day oral administration of Q + D [61]. The study was not powered to test efficacy and did not include a control group, but the observed effects of the senolytic cocktail were confirmed in vivo, as the expression of p16^INK4a^ and p21^Cip1^ was shown to respond to treatment with Q + D in immunodeficient mice transplanted with fat from obese patients [64]. Interestingly, in diabetic patients with CDKD, expression of senescence of p21^Cip1^ and other senescence biomarkers was shown to be increased in the tubular epithelium, while the levels of the corresponding p21 protein in urine were found to be elevated [65]. More studies are needed to establish the value of p21^Cip1^ as a diagnostic biomarker reflecting disease progression in CDKD as well as a response biomarker for monitoring the effects of senolytic interventions in metabolic ARDs.

The study by Hickson et al. (2019, 2020) also found significant reductions in MMP-2, MMP-9 and MMP-12 in the plasma of diabetic patients, as well as a reduction in plasma IL-1α and IL-6 levels after Q + D treatment [61], which might be further attributed to the senolytic effects of the tested intervention.

In patients with early AD and MCI, target engagement of Q + D combination was assessed by measuring the expression of senescence biomarkers p16^INK4a^ and p21^Cip1^ in T lymphocytes and SASP factors in blood [60] and cerebrospinal fluid (CSF) and plasma [59]. The intermittent administration of Q + D for 12 weeks in MCI patients did not affect the expression of p16^INK4a^ and p21^Cip1^ in T lymphocytes, but it decreased blood levels of TNF-α by 15.5% [60]. Interestingly, the study indicated some benefits of senolytic intervention on cognitive and mobility outcomes in subjects with lower MoCA scores, which are reflective of worse global cognitive function, showing a significant reduction in TNF-α and a tendential decrease in other SASP factors as a result of Q + D treatment [60]. Mobility outcomes, such as dual-task gait speed and dual-task cost of stride length, also insignificantly improved in the study [60], demonstrating the potential of the Q + D combination to modulate relevant clinical outcomes.

A 12-week study testing the effects of Q + D in mild AD patients did not find beneficial changes in mean Montreal Cognitive Assessment (MoCA) scores or Clinical Dementia Rating (CDR); however, it demonstrated some improvement on a neuropsychological test of verbal learning and memory after 12 weeks of intermittent use of a senolytic cocktail (Table 2) [59]. The study also demonstrated a marked increase in IL-6 in CSF and some decline in the level of this SAPS factor in blood after treatment, which was attributed to the senolytic effect of the intervention [59]. Baseline-to-post-treatment analysis identified decreases in multiple SASP factors, which were more prominent in CSF than in blood [59]. A follow-up study further explored changes in the levels of SASP factors in various biological fluids, as well as gene expression changes in peripheral blood mononuclear cells obtained from participants in the study by Garbarino et al., 2025 [66]. Targeted cytokine and chemokine analyses additionally revealed increases in plasma fractalkine and MMP-7 from baseline to post-treatment and identified downregulation of inflammatory genes, including *FOS*, *FOSB*, *IL1β*, *IL8*, *JUN*, *JUNB*, and *PTGS2*, while the levels of amyloid β and tau proteins in CSF and profiles of urinary metabolites remain unchanged. Thus, the current evidence provides exploration into treatment responses of SASP factors and senescence biomarkers, which may be important in informing trial design and outcome selection for senolytic studies.

### 4.3. Ongoing and Upcoming Studies

Using the database www.clinicaltrials.gov, we identified 16 ongoing or planned clinical trials defined mainly as phase 1 or 2 RCTs, which aim to investigate the potential of fisetin, quercetin and/or Q + D to modulate senescence biomarkers and SASP factors in human subjects with ARDs (Table 3). The target populations include patients with musculoskeletal disorders and cardiovascular and neurodegenerative diseases, as well as geriatric syndromes and obesity (Table 3). The upcoming RCTs NCT04685590 and NCT02848131 are based on published pilot studies assessing the effects of Q + D and will enroll older subjects with MCI and CDKD, respectively. The evaluation of nutraceuticals in musculoskeletal disorders is supported by translational evidence from animal models of aging [40,45,46], experiments in human [67] and mouse [68] chondrocytes and animal models of osteoarthritis [68,69]. The majority of the trials were designed based on clinical outcomes (e.g., physical function, symptoms, disease progression) and/or safety characteristics as primary outcomes, while some relevant senescence biomarkers and SASP factors were included as secondary outcomes. The expression of p16^INK4a^ in various cell types is the most frequently assessed biomarker (NCT06431932; NCT04733534; NCT06133634; NCT05416515; NCT04313634; NCT05422885; NCT04685590; NCT0474907253), followed by the expression of p21^Cip1^ (NCT06431932; NCT06133634). Only one study (NCT04733534) enrolling frail childhood cancer survivors was designed with the expression of the senescence biomarker p16^INK4a^ in T lymphocytes identified as the main study outcome. With regard to SASP, all protocols intend to measure these factors in various biological materials, mainly as secondary or other outcomes, in humans with ARDs.

We identified several upcoming clinical trials that characterize the senotherapeutic potential of fisetin. The rationale for the use of fisetin in geriatric syndromes has been supported by evidence from preclinical research completed in aged and progeroid mice (Table 1). Among the planned fisetin trials, four are placebo-controlled, while others explore alternative designs, including a dose comparison study (e.g., low- vs. high-dose fisetin in NCT04770064), combination with pharmaceutical agents (e.g., fisetin in combination with losartan) (NCT04815902) or integration with lifestyle interventions (e.g., mind–body program in NCT05482672). The dose of 20 mg/kg/d will be most commonly used. Interestingly, a planned study investigating the effects of fisetin in multimorbidity (NCT06431932) has been designed with levels of soluble urokinase plasminogen activator receptor (suPAR) recognized as a primary study outcome. SuPAR, the soluble counterpart of urokinase plasminogen activator receptor, is found in the circulation at various levels. Together with its parent molecule, cell surface uPAR, it exhibits a similar structure and extracellular functional roles facilitating fibrinolysis, cellular adhesion, and migration [70]. Recognized to increase during the states of immune activation, suPAR was also shown to be expressed on accumulating SCs and has been proposed as a SASP factor [12]. One study, NCT06431932, involves senescence biomarkers and SASP factors as secondary endpoints, which is of major interest not only with regard to the mechanistic effects of fisetin in humans but also potentially clinical validation of senescence biomarkers during aging. The other SASP factors are intended to be tested as secondary or exploratory outcomes in the upcoming studies, and their choices seem to be largely justified by their links to the pathobiology of senescence, as well as to specific ARDs.

The upcoming studies with Q + D follow a similar dosing strategy to the completed human studies and compare the studied intervention either to a placebo (NCT04685590, NCT02848131) or to exercise (NCT05653258).

## 5. Discussion

Identifying non-pharmacological strategies targeting various mechanisms involved in senescence is an important objective of aging research. The dietary bioactives quercetin and fisetin have been increasingly acknowledged for their protective effects on cellular aging [9,12,71]. In the reviewed animal models, the senolytic action of these nutrients was most frequently confirmed by their ability to limit the expression of *p16^INK4a^* and *p21^Cip1^*, their SA-β-gal activity in organs and tissues and their senomorphic action by changes in the expression of IL-1α, IL-1β, IL-6, MCP-1 and TNF-α (Table 1). Similar biomarkers were also commonly applied in completed and upcoming clinical studies (Table 2 and Table 3). Based on the dynamics of changes in selected biomarkers and certain clinical improvements, both bioactives are of great promise for the management of ARDs. At the same time, clinical evidence supporting their efficacy is yet to come.

The doses of fisetin (20 mg/kg/d) and quercetin (1000–1250 mg/d) used in clinical trials far exceed those that can be reached by dietary intake. These choices are substantiated by preclinical studies showing the senolytic effect of fisetin at concentrations between 5 μM and 20 μM, depending on the cell type [39]. For quercetin, a dose of 10 μM to 20 μM has been identified as the lowest effective concentration for senolysis, respectively, in aged HUVEC cells and adipocytes [18,72]. Furthermore, clinical translation of fisetin has been hampered by its poor water solubility (10.4 μg/mL) [73] and low bioavailability, as administration of 100 mg/kg of unformulated fisetin to mice resulted in an absolute bioavailability of 7.8% [74]. A recent study in humans showed that oral intake of 1000 mg of fisetin by healthy volunteers resulted in a maximum plasma concentration of only 9.97 ng/mL (0.035 M), with a tmax of 0.88 h [75]. Similar to fisetin, quercetin is characterized by poor oral bioavailability as a consequence of low water solubility (approximately 10 μg/mL for aglycone), premature degradation, and extensive first-pass metabolism [76]. The peak plasma concentrations of quercetin in human subjects were reported to reach 5 to 10 μM after the administration of a single dose of 500 mg [77]. To enhance the bioavailability of both nutrients, various strategies are being explored, including encapsulation into lipid vesicles or nanoparticles, complexation with cyclodextrins, or self-nano-emulsifying drug delivery systems [78]. Identification of the optimal delivery system can potentially help to optimize dosing and achieve better clinical outcomes.

Animal studies have shown that fisetin and quercetin, alone or in combination with dasatinib, have a favorable safety profile, even when taken at high doses and for longer periods [51,79]. Available human studies evaluating the safety of Q + D in ARDs are limited but report only mild side effects, such as diarrhea, shortness of breath, skin irritation, gastrointestinal discomfort and headache [59,60,62]. Dasatinib alone appears to be well tolerated, displaying a similar safety profile as Q + D treatment [80]. Information about the safety of long-term intake of high doses of fisetin in humans is limited. A trial in men with Gulf War Illness who received up to 800 mg of fisetin daily for one month reported only mild side effects, including dizziness, fatigue, nausea, headache and gastrointestinal discomfort [81]. Importantly, since both fisetin and quercetin are recognized as potent antioxidants, their long-term use in humans should be carefully monitored due to the potential disruption of the body’s natural redox balance, interfering with redox-sensitive signaling pathways [82]. Upcoming studies will provide more insight into the safety of these bioactives.

The analysis of in vivo studies identified a potential modulatory genetic effect, as well as gender and age, on the outcomes of nutritional senolysis. For example, in contrast to other animal models that tested the effects of fisetin on senescence, the study using UM-HET3 mice did not show any changes in SC burden in the liver, kidney and brain after either daily or intermittent supplementation with fisetin and did not increase the lifespan of the mice [52]. Compared with inbred C57BL/6 mice used in other studies, outbred UM-HET3 mice are more genetically heterogeneous and are believed to yield results that are more generalizable to clinical research [83]. This model has also been identified as a critical component of a program that is recommended to be followed to ensure that the effectiveness of lifespan-extending compounds is not limited by genetic background [83]. Therefore, the potential effects of genetic factors on the senotherapeutic effects of fisetin warrant further studies.

In addition, sex-specific and age-dependent responses could have contributed to the variability in findings between animal studies. Baier et al. (2025) reported that Q + D had differential effects on the expression of *p16^INK4^* and *p21^Cip1^* in the hippocampal tissue of 22-month-old male and female C57BL/6 mice, while a reduction in SA-β-gal+ cells in the hippocampal tissue was demonstrated only in males [41]. Interestingly, the effect of sex on the outcomes of senolytic interventions has already been reported in clinical research. A recent randomized, double-blind, placebo-controlled clinical trial involving 97 coronary artery disease patients who received either 500 mg of quercetin twice daily or a placebo for two days before undergoing bypass surgery showed a strong sex-dependent effect on the senescence biomarkers p21 (*CDKN1A*) and β-galactosidase (*GLB1*) p21^Cip1^, as those were significantly downregulated in male but not female arterial cells obtained from arterial segments [84]. While the exact reason for observing sexual dimorphism during nutritional senolysis is not clear, differences in the overall level of SC burden and drug metabolism may offer a possible explanation [85]. Additionally, loss of estrogens may have facilitated the processes of cellular senescence, as estrogen-induced signaling and gene regulation change over the course of aging [85] due to shifts in expression of the estrogen receptor (ER)α, ERβ and G-protein coupled ER [86,87] and epigenetic modifications associated with estrogen loss [86,87,88].

Furthermore, Q + D treatment was shown to exert an age-dependent response with reduced p16^INK4a^ expression in the intervertebral discs of 14-month-old mice but not those of 18-month-old mice, suggesting that Q + D may be effective in slowing the process of senescence and disc degeneration when administered during the early stages of the disease process but not when disc degeneration is advanced [46]. Interestingly, the study by Dungan et al. (2022), subjecting both old and young C57BI/6J mice to Q + D treatment with subsequent induction of muscle tissue damage, showed that a significant decrease in the abundance of SA-β-gal+ cells was accompanied by improved proliferation of myogenic progenitor cells only in old mice, suggesting age-dependent beneficial effects of senolytics on muscle regeneration [42]. Defining the optimal window of opportunity remains an important task for nutritional senolysis, as together with an improved understanding of sex-specific differences, it can significantly facilitate advances to clinical trials.

Another important concern related to the clinical introduction of those nutrients is linked to their potential interaction with drugs, as both fisetin and quercetin have been shown to inhibit several CYP450 enzymes [12,89,90,91]. Namely, quercetin and its metabolites (quercetin-30-sulfate, quercetin-3-glucuronide, isorhamnetin, and isorhamnetin-3-glucuronide) showed weak inhibitory effects on CYP2C19 and CYP3A4, but they did not affect CYP2D6 activity [89]. Since CYP2C19 is involved in the metabolism of proton pump inhibitors, antiplatelet drug clopidogrel, antidepressants and benzodiazepines, their pharmacokinetics can be potentially affected. Fisetin, on the other hand, was shown to induce concentration-dependent inhibition of CYP2C8 [91], which is involved in the metabolism of anti-diabetic, antimalarial and some cancer medications [92]. Furthermore, fisetin showed moderate inhibitory effects on CYP2D6, which is involved in the metabolism of beta blockers, and weak inhibitory effects on CYP3A4, which is known to metabolize calcium channel blockers [90]. Since an aging population frequently uses multiple drugs to manage ARDs, possible drug–nutrient interactions should be taken into account.

We identified only only a few completed clinical trials: all tested the Q + D combination, but none were designed or powered to assess the efficacy. Interestingly, administration of Q + D for three days in CDKD caused a significant reduction in most of the tested biomarkers [61], while longer intermittent administration in IPF and AD/MCI patients was associated with more discrepant findings; however, a reduction was shown in at least one senescence marker (e.g., *p16^INK4a^* and *p21^Cip1^* in T-lymphocytes or IL-6, TNF-α and MMPs in blood) [59,60,62]. The design of studies aiming to test the effects of nutritional senolysis faces the challenge of selecting biomarkers that would most reliably reflect the mechanistic effects of the interventions and ideally would also be linked to the progression of the disease. Recently, it has been suggested that combinations of markers, including p16^INK4a^ and p^21Cip1^ expression, SA-β-gal activity and SASP factors should be used to unequivocally identify SCs and assess potential treatment effects [93]. This approach is supported by the lack of specificity of indicated biomarkers, as both SA-β-gal activity and *p16^INK4a^* expression have been observed in non-senescent cells, including young cells, neurons, and developing embryos [6,93]. Across the reviewed evidence, various SASP factors are measured, with common emphasis on IL-1α, IL-1β, IL-6, IL-8, IL-10, TNF-α and MCP-1. The use of a broad variety of biomarkers can be partially explained by the still incomplete understanding of the biological nature of aging [1], including heterogeneity in SC populations and their tissue-specific accumulation [39,94]. Furthermore, many SASP components also participate in physiological processes unrelated to senescence, making it difficult to attribute observed changes solely to SC burden [12,28]. To overcome those challenges, integrative and multimodal approaches, which involve assessment of multiple clinical, physiological and biochemical indices, were proposed [95,96]. The systems PhenoAge and GrimAge were trained on panels of age-associated molecular and physiological biomarkers and appear to be effective in estimating biological age, but their validation as efficacy biomarkers in studies assessing effects of nutritional senolysis remains to be investigated.

## 6. Conclusions

In conclusion, this literature review highlighted the senolytic potential of fisetin and quercetin in animal models of aging and progeroid syndromes. Although results of human studies are encouraging, the field remains in its infancy, and the current available human evidence remains too limited to make definitive conclusions regarding the efficacy and long-term safety of fisetin and quercetin. Future research will help to answer these questions and support the development of target engagement assays and integrative biomarkers that can be used to monitor the effects of nutritional senolysis in humans.

## Figures and Tables

**Table 2:** Studies exploring the potential of quercetin in combination with dasatinib to modulate senescence biomarkers in human subjects.

Study Design	Condition	Sample	Dosage	Senescence Biomarker	Results	Ref.
**Single-arm, open-label, phase 1 study**	Chronic diabetic kidney disease	n = 9, mean age 69 y, M/F: 7/2	1000 mg/d Q orally + 100 mg/d D for 3 days	p16^INK4a^, p21^Cip1^ and SA-B-gal+ cells in adipose tissue and epidermis; IL-1α, -1RA, -2, -6, MMP-2, -9, -12 and GM-CSF in plasma; measured 11 days after intervention	Q + D significantly reduced p16^INK4a^, p21^Cip1^ and SA-β-gal+ cells in adipose tissue in grouped analysis; p16^INK4a^ and p21^Cip1^ in epidermis were not statistically decreased; Q + D reduced plasma IL-1α, -2, -6, and -9 and MMP 2, -9, and -12.	[61]
**Single-arm, open-label, pilot study**	Stable IPF	n = 14, mean age 70.8 y; M/F: 2/12	1250 mg/d Q + 100 mg/d D orally for 3 consecutive days for three weeks	Serum IL-1α, -1β, -1RA, -2, -3, -4, -5, -6, -8, -9, -10, -18, CCL5, CXCL10, MCP1, MIP-1α, -1β, -1, -2, -3, -7, -8, -9, -10, -12, -13, GCSF, GM-CSF, PAI-1, PDGF-AA, PGF-BB, TGF-α, TIMP1, -2, -4, and TNF-α.	Senescence and SASP measures: Q + D nonsignificantly reduced IL-7 and TIMP2; no effect on all other markers of SASP. Clinical measures: Q + D significantly improved physical function measures (6MWD, 4 m gait speed, chair stands and SPPB), while there was no effect on pulmonary function (FVC and FEV1), quality of life and fatigue.	[62]
**Single-arm, open-label, phase I study**	Early-stage AD/MCI	n = 5, mean age 76 y, M/F: 3/2	1000 mg/d Q + 100 mg/d D orally for 2 consecutive days every 2 weeks for 12 weeks	IL-6, -10 -17E, -21, -23, -17A/F, -17D,-31, VEGF, MCP-2, MIP-1α and MIP-1β in plasma; IL-6, -17A and MIP-1α in CSF	Senescence and SASP measures: In plasma Q + D significantly decreased plasma IL-10, -17E, -21, -23, 17A/F, -17D, VEGF, MCP-2, MIP-1α and MIP-1B levels; no effect on IL-6. In CSF, Q + D significantly decreased IL-17A and MIP-1a levels and increased IL-6. Clinical measures: Q + D significantly decrease HVLT-R immediate recall, while there was no effect on MoCA and CDR.	[59]
**Single-arm, open-label, pilot study**	Early-stage AD/MCI	n = 12, mean 77 y, M/F: 5/7	1250 mg/d Q + 100 mg/d D orally for 2 consecutive days every 2 weeks for 12 weeks	Serum IL-1α, -6, -7, -8, -10, -18, CCL11, CCL17, -19, -2, -22, CXCL1, -10, -5, GDF-15, MMP-1, -2, -3, -7, -8, -9, -10, PDGF-AA, PDGF-AB/BB, PDGF-BB, TIMP-1, -2, -4, TNF-α and VCAM	Senescence and SASP measures: Q + D significantly reduced serum TNF-α; insignificantly reduced serum IL-6 and had no effect on other senescence and SASP biomarkers. Clinical measures: Q + D insignificantly improved MoCA, TMT B-A, dual-task gait speed, dual-task cost of gait speed, dual-task cost of stride length, and stride length. Q + D did not affect SPPB, max grip strength, normal walking stride length and normal walking gait speed.	[60]

**Table 3:** Ongoing and upcoming studies exploring the potential of quercetin and fisetin to modulate senescence biomarkers in human subjects.

NCT	Design	Condition	N	Age	Intervention	Dosing	Senescence Outcome	Status
**Fisetin**								
**Geriatric Syndrome**								
**06431932**	RCT Phases 1 and 2	Multimorbidity	40	≥65	FIS or placebo	20 mg/kg/d orally for 2 consecutive days	Primary Outcome: Plasma suPAR Secondary Outcomes: Expression of p16^INK4a^, p21^Cip1^ and SA-B-gal in immune cells in blood, skin and adipose tissue Plasma SASP factors and inflammation markers (specific biomarkers not specified)	Not yet recruiting. Study completion estimated for 2034.
**04733534**	Open-label Phase 2	Frailty in childhood cancer survivors	60	≥18	FIS or Q + D	20 mg/kg/d orally for 2 consecutive days, repeated after one month	Primary Outcome: p16^INK4a^-positive T-lymphocytes in blood	Recruiting. Study completion estimated for 2026.
**Cardiovascular**								
**06133634**	RCT Phases 1 and 2	Endothelial dysfunction and arterial stiffness	70	≥65	FIS or placebo	20 mg/kg/d orally for 3 consecutive days, repeated after two weeks	Other Outcomes: p16^INK4a^ and p21^Cip1^ positive endothelial cells, p16^INK4a^-positive T-lymphocytes in blood, and circulating pro-inflammatory cytokines and chemokines (specific biomarkers not specified)	Recruiting. Study completion estimated for 2027.
**06399809**	RCT Phase 2	Peripheral artery disease	34	≥50	FIS or placebo	20 mg/kg/d orally for 2 consecutive days, repeated after 12 days	Other Outcomes: IL-1α, -6, -8, MCP-1 and GDF-15 expression in adipose tissue and IL-6 gene expression in gastrocnemius muscle and adipose tissue	Recruiting. Study completion estimated for 2027.
**Musculoskeletal Disorders**								
**04770064**	RCT Phases 1 and 2	Knee osteoarthritis	60	34–80	Low-dose FIS or high-dose FIS or placebo	LD: 100 mg/d orally daily for 90 days HD: 20 mg/kg/d orally for 2 consecutive days, repeated after one month	Secondary Outcome: Serum MMP3 concentration	Withdrawn (funding not provided).
**04313634**	RCT Open-label Phase 2	Postmenopausal bone loss	74	≥60	Q + D or FIS or control (no intervention)	20 mg/kg/d orally for 3 consecutive days, repeated each month for 5 months	Secondary Outcomes: p16^INK4a^-positive T-lymphocytes in blood and plasma IL-6 and -8 and MCP-1 expression in blood	Completed 2023; results not retrieved.
**05416515**	Open-label Phase 2	Carpal tunnel syndrome	40	21–80	FIS	20 mg/kg/d orally for 2 consecutive days, repeated after one month	Secondary Outcomes: p16^INK4a^, IL-6, -15, TNF-α and PAI-1 expression in blood	Active; not recruiting. Study completion estimated for 2025.
**05025956**	RCT Phases 1 and 2	Femoroacetabular impingement	68	18–80	FIS or placebo	20 mg/kg/d orally for days 1 and 2 before surgery, repeated every month for 2 consecutive days for 2 months	Secondary Outcomes: Concentrations of senescence and SASP markers found in serum (specific biomarkers not specified)	Active; not recruiting. Study completion estimated for 2024.
**04815902**	RCT Phases 1 and 2	Knee osteoarthritis	100	40–85	FIS in combination with active Losartan or with a Losartan placebo or placebo	20 mg/kg/d orally for 2 consecutive days one month before BMAC injection, 2 consecutive days immediately before BMAC injection, and 2 consecutive days after BMAC injection, repeated every month over three months	Secondary Outcomes: IL-1β, -6, -15, -1α, -8, -18, CCL5, CXCL10, GDF15, MCP-1, MMP-1, -2, -9, -10, TIMP1, TIMP2, TNF-α, VEGF expression in peripheral blood plasma	Active; not recruiting. Study completion estimated for 2025.
**05482672**	RCT Phases 2 and 3	Knee osteoarthritis Depression Obesity	120	≥40	FIS + mind body program or placebo + usual health-care education	20 mg/kg/d orally for 2 consecutive days, repeated after one month	Secondary Outcomes: Serum IL-4 and IL-17	Withdrawn (due to lack of funding).
**Quercetin**								
**Geriatric Syndromes**								
**04733534**	Open-label Phase 2	Frailty in childhood cancer survivors	60	≥18	Q + D or FIS	1000 mg/d Q + 100 mg/d D orally for 3 consecutive days, repeated after one month	Primary Outcome: p16^INK4a^-positive T-lymphocytes in blood	Recruiting. Study completion estimated for 2026.
**Musculoskeletal Disorders**								
**04313634**	RCT Open-label Phase 2	Postmenopausal women	74	≥60	Q + D or FIS or control (no intervention)	1000 mg/d Q + 100 mg/d D orally for 2 consecutive days repeated each month for 5 months	Secondary Outcomes: p16^INK4a^-positive T-lymphocytes in blood Plasma IL-6, -8 and MCP-1 gene expression	Completed 2023; results not retrieved.
**Neurodegenerative Diseases**								
**05422885**	Open-label pilot study	Older adults with slow gait speed and MCI	12	≥65	Q + D	1250 mg/d Q + 100 mg/d D orally for 2 consecutive days every two weeks for 3 months	Secondary Outcome: p16^INK4a^-positive T-lymphocytes in blood IL-1α, -6, MMP-9 and MMP-12 in blood and urine	Completed 2024; results not published.
**04685590**	RCT Open-label Phase 2	Older adults with MCI or early-stage AD	48	≥60	Q + D or placebo	1000 mg/d Q + 100 mg/d D orally for 2 consecutive days every 2 weeks for three months	Secondary Outcomes: p16^INK4a^-positive T-lymphocytes and IL-1α, -1β, -2, -6, MMP expression in blood	Active; not recruiting. Study completion estimated for 2029.
**Obesity**								
**05653258**	RCT, Phases 2 and 3	Obese older adults	40	≥65	Exercise or calorie restriction or Q + D, or placebo	1000 mg/d Q + 100 mg/d D orally for 3 consecutive days every month for two months	Secondary Outcomes: SASP in adipose tissue (specific biomarkers not specified)	Recruiting. Study completion estimated for 2027.
**Kidney Disease**								
**02848131**	RCT Open-Label Phase 2	Chronic kidney disease and diabetes mellitus	30	40–80	Q + D or no intervention	1000 mg/d Q + 100 mg/d D orally for three consecutive days	Secondary Outcomes: Proportion of SCs in skin, fat, and/or blood (senescent biomarker not specified)	Enrolling by invitation. Study completion estimated for 2025.

## Data Availability

No new data were created or analyzed in this study. Data sharing is not applicable to this article.

## Abbreviations

6MWD	6-min walk distance
AD/MCI	Alzheimer’s disease or mild cognitive impairment
APO E	Apolipoprotein E
APO E KO	Apolipoprotein E knockout
ARD	Age-related disease
Bacl2	Barium chloride
Bax	Bcl-2-associated X protein
Bak	Bcl-associated killer
BCL-W	B-cell lymphoma-W
Bcl-2	B-cell lymphoma 2
Bcl-xl	B-cell lymphoma-extra large
BMAC	Bone Marrow Aspirate Concentrate
CABG	Coronary Artery Bypass Graft
CCL	C-C motif ligand
CD3	Cluster of differentiation 3
CD3+ T cells	T cells expressing CD3
CD8+ T cells	CD8-positive T lymphocytes
CDK	Cyclin-dependent kinase
CDR	Clinical Dementia Rating
CDKD	Chronic diabetic kidney disease
CSF	Cerebrospinal fluid
CYP450	Cytochrome P450
CXCL	C-X-C motif chemokine ligand
D	Dasatinib
Q + D	Quercetin + Dasatinib
DDR	DNA damage response
Ercc1^−/Δ^	Excision repair cross-complementation group 1 mutant mouse
F	Female
FEV1	Forced expiratory volume in 1 s
FIS	Fisetin
FJ OA	Facet Joint Osteoarthritis model
FDA	Food and Drug Administration
G-CSF	Granulocyte-colony stimulating factor
GM-CSF	Granulocyte–macrophage colony-stimulating factor
HFD	High-fat diet
HVLT-R	Hopkins Verbal Learning Test—Revised
INF-γ	Interferon gamma
IPF	Idiopathic pulmonary fibrosis
IL	Interleukin
M	Male
MCP	Monocyte Chemoattractant Protein
MMP(s)	Matrix metalloproteinase(s)
MIP(-1α)	Macrophage Inflammatory Protein (1 alpha)
MoCA	Montreal Cognitive Assessment
NF-κB	Nuclear factor kappa B
NK cells	Natural killer cells
NCT	National Clinical Trial
OS	Oxidative stress
PAI-1	Plasminogen activator inhibitor-1
pgWAT	Perigonadal white adipose tissue
PI3K/AKT	Phosphoinositide 3-kinase/protein kinase B signaling pathway
Q	Quercetin
RB	Retinoblastoma protein
ROS	Reactive oxygen species
RCT	Randomized Control Trial
SA-β-gal	Senescence-associated β-galactosidase
SAMP10	Senescence-accelerated mouse-prone 10
SC	Senescent cell
SNEDDS	Self-nano-emulsifying drug delivery system
SPPB	Short physical performance battery
SASP	Senescence-associated secretory phenotype
suPAR	Soluble urokinase plasminogen activator receptor
TGF-α	Transforming growth factor alpha
TGF-β	Transforming Growth Factor Beta
Tert	Telomerase reverse transcriptase knockout mice
TNF-α	Tumor necrosis factor alpha
UM-HET3	Four-way cross genetically heterogeneous mouse line
VCAM	Vascular cell adhesion molecule-1
VEGF	Vascular endothelial growth factor
vWAT	Visceral white adipose tissue
W	With
